# Editorial: Recent advances in understanding the genetics of immunological disorders

**DOI:** 10.3389/fgene.2024.1530187

**Published:** 2024-12-02

**Authors:** Che Kang Lim, Hassan Abolhassani, Jinqiao Sun

**Affiliations:** ^1^ Department of Clinical Translational Research, Singapore General Hospital, Singapore, Singapore; ^2^ Medicine Academic Clinical Programme (ACP), DUKE-NUS Medical School, Singapore, Singapore; ^3^ Division of Immunology, Department of Medical Biochemistry and Biophysics, Karolinska Institutet, Stockholm, Sweden; ^4^ Research Center for Immunodeficiencies, Pediatrics Center of Excellence, Children’s Medical Center, Tehran University of Medical Sciences, Tehran, Iran; ^5^ Department of Clinical Immunology, National Children’s Medical Center, Children’s Hospital of Fudan University, Shanghai, China

**Keywords:** immunological diseases, genetics, primary immumunodeficiencies, autoimmune diseases, allergy diseases, next-generation sequencing (NGS)

Immunological disorders are conditions arising from immune system dysfunction, encompassing primary immunodeficiency syndromes, inborn errors of immunity, autoimmune diseases, asthma, allergies, lymphoproliferative and autoinflammatory syndromes. These disorders compromise the body’s defense against pathogens and may cause exaggerated chronic inflammation responses and tissue damage. Owning clinical and genetic heterogeneity, immunological disorders exhibit a broad phenotypic spectrum, and distinct genotypes can produce overlapping clinical presentations. Additionally, the same mutation in immune-related genes within the same family can appear across multiple disorders, making accurate diagnosis and effective management complex and challenging.

In the past decade, remarkable advancements in genomics and sequencing technologies have revolutionized our understanding of candidate genes in immunological disorders. With new tools such as next-generation sequencing (NGS), allowing for in-depth genomic analysis, substantial progress has been made in identifying disease-causing genes associated with rare monogenic conditions, especially immunodeficiency syndromes (Vorsteveld et al., 2021; Conley and Casanova, 2014). These discoveries have yielded critical insights into the molecular mechanisms driving immune dysfunction, revealing how specific genetic mutations can impact immune pathways and trigger various immunological disorders. Importantly, the identification of these disease-causing genes has also paved the way for discovering novel biomarkers, which serve as valuable tools in clinical settings by enhancing diagnostic precision and aiding in the prediction of disease progression (Yazdanpanah and Rezaei, 2024; Peng and Kaviany, 2023). These biomarkers are instrumental in tailoring targeted therapeutic strategies, allowing for more effective and personalized treatment approaches. This genomic knowledge has not only enhanced our understanding of rare inborn errors of immunity but also laid the foundation for ongoing innovations in the diagnosis, management, and treatment of frequent complex immunological conditions. These findings shed insights into disease mechanisms and facilitated the identification of new biomarkers that can help in diagnosis, prognosis, and development of novel clinical therapeutic approaches.

This Research Topic presents eleven groundbreaking articles exploring the complex molecular genetics of immunological diseases. This compilation provides a comprehensive analysis of recent advancements in identifying the genetic and molecular mechanisms of immune-related disorders, including autoimmune diseases, allergies, and primary immunodeficiencies. It emphasizes the growing role of genetic insights, shared biological pathways, and biomarkers in revolutionizing clinical care. These studies collectively highlight the transformative potential of integrating innovative diagnostic techniques and precision medicine into the management of common immune disorders with high public health burdens.

In this Research Topic, significant findings span a range of conditions in autoimmune diseases, showcasing the interplay of genetic heterogeneity and shared pathways. For systemic sclerosis (SSc), Hanson et al. validated HLA Class II associations while uncovering a novel HLA Class I haplotype, HLA-B44:03-HLA-C16:01, linked to disease heterogeneity. This discovery, alongside the genetic interplay between killer cell immunoglobulin-like receptors (KIRs) and HLA ligands, offers critical insights into SSc’s molecular mechanisms. Similarly, in rheumatoid arthritis (RA), Wen et al. identified 82 shared risk genes with other autoimmune conditions, such as multiple sclerosis (MS) and type 1 diabetes (T1D). These findings highlight overlapping disease mechanisms and the potential for cross-disease therapeutic strategies. Additionally, Ou et al. highlighted *TWIST1* gene as a key factor in ulcerative colitis (UC), while Chng et al. suggested that tear *S100A4* emerged as a biomarker for predicting thyroid eye disease (TED) in Graves’ orbitopathy (GO). Fan et al. revealed that *CLEC16A* genetic variants, notably, the A allele of rs6498169 and the G allele of rs7200786, are associated with autoimmune diseases and also confer susceptibility to Parkinson’s disease (PD) in Han Chinese. These findings emphasize the potential shared pathways between neurodegeneration and immune dysfunction. Finally, Yeo et al. reviewed articles on systemic lupus erythematosus (SLE) and discussed the implications of causal variants in both polygenic and monogenic forms of the disease. It suggests an age-based sequencing strategy to improve diagnostics and management, addressing genetic disparities and tailoring approaches to patient-specific factors.

Investigation in the field of allergy has also revealed important molecular mechanisms in the current Research Topic, particularly in allergic rhinitis (AR) and asthma. Sun et al. identified *ZNF667-AS1* as a key mediator in disease progression, driven by pollen-induced methylation and type 2 inflammatory pathways. The findings provide new avenues to mitigate disease progression in allergic disorders, reflecting the broader potential of genetic research to inform tailored treatments.

Some recent studies in the field of primary immunodeficiency or inborn errors of immunity featured the diagnostic utility of linking clinical phenotypes to underlying genetic defects. In common variable immunodeficiency (CVID), Cunningham-Rundles et al. examined a cohort of 405 patients and highlighted the effectiveness of phenotype-guided genetic diagnostics, particularly in cases involving autoinflammation or autoimmunity. This approach helps pinpoint the specific genetic mutations responsible for these predominantly antibody-deficient conditions, improving the accuracy of diagnoses, and paving the way for more targeted interventions.

Three case reports on rare disorders in the Research Topic further illustrate the importance of advanced genetic testing. Li et al. reported a child with COPA syndrome presented with interstitial lung disease and neuromyelitis optica spectrum disorder (NMOSD), underscoring the utility of genomic sequencing in diagnosing rare phenotypes. Deng et al. presented a case involving severe combined immunodeficiency (SCID) caused by compound heterozygous mutations in the *DCLRE1C* gene. Advanced genetic testing revealed a unique manifestation of rubella virus-induced cutaneous granulomas, which had not been previously associated with SCID. Additionally, Chen et al. identified novel variants in *TCRIG1* and *CLCN7* genes, contributing to a deeper understanding of genotype-phenotype correlations in osteopetrosis. These findings underscore the importance of further research into the genetic mechanisms and potential treatments for these complex immune-related disorders.

In conclusion, this Research Topic highlights the translational impact of genetic research on the diagnosis, treatment and prognosis estimation of immunological disorders. By uncovering novel genetic associations, unique biomarkers, and shared pathways, these studies provide a strong foundation for the development of targeted/personalized medicine. Integrating these insights into clinical practice holds significant potential to improve patient outcomes, especially in managing the complex and heterogeneous nature of immune conditions. As genetic research continues to evolve, it promises to unlock new opportunities for individualized care and therapeutic innovations. We highly believe that the compilation of publications within this Research Topic offers a valuable snapshot of the current focus and progress in the field of genetic research on human immunological disorders.

## References

[B1] Conley M. E. Casanova J. L. (2014). Discovery of single-gene inborn errors of immunity by next generation sequencing. Curr. Opin. Immunol. 30, 17–23. 10.1016/j.coi.2014.05.004 24886697 PMC4198453

[B2] Peng X. Kaviany S. (2023). Approach to diagnosing inborn errors of immunity. Rheumatic Dis. Clin. N. Am. 49 (4), 731–739. 10.1016/j.rdc.2023.06.001 37821192

[B3] Vorsteveld E. E. Hoischen A. van der Made C. I. (2021). Next-generation sequencing in the field of primary immunodeficiencies: current yield, challenges, and future perspectives. Clin. Rev. Allergy Immunol. 61 (2), 212–225. 10.1007/s12016-021-08838-5 33666867 PMC7934351

[B4] Yazdanpanah N. Rezaei N. (2024). The multidisciplinary approach to diagnosing inborn errors of immunity: a comprehensive review of discipline-based manifestations. Expert Rev. Clin. Immunol. 20 (10), 1237–1259. 10.1080/1744666X.2024.2372335 38907993

